# Tumour-infiltrating lymphocyte therapy in melanoma: ready for prime time?

**DOI:** 10.1038/s41416-026-03350-z

**Published:** 2026-02-18

**Authors:** Rachel Woodford, Paul Lorigan, Deemesh Oudit, Ahmed Abdulgawad, Fiona Thistlethwaite, Kok Haw Jonathan Lim

**Affiliations:** 1 Advanced Immunotherapy & Cell Therapy Team, Department of Medical Oncology, The Christie NHS Foundation Trust, Manchester, UK; 2 National Health and Medical Research Council Clinical Trials Unit, University of Sydney, Camperdown, NSW Australia; 3 Melanoma Team, Department of Medical Oncology, The Christie NHS Foundation Trust, Manchester, UK; 4 Division of Cancer Sciences, Faculty of Biology, Medicine and Health, The University of Manchester, Manchester, UK; 5 Plastic Surgery Team, Department of Surgery, The Christie NHS Foundation Trust, Manchester, UK; 6 Cellular Therapies & Early Phase Trials Team, Department of Haematology, The Christie NHS Foundation Trust, Manchester, UK

**Keywords:** Cancer immunotherapy, Melanoma, Immunotherapy

## Abstract

Tumour infiltrating lymphocyte (TIL) therapy offers the potential for durable clinical benefit in select patients with advanced melanoma, especially after progression on treatment with immune checkpoint inhibitors and/or targeted therapies. The 2024 FDA approval of Lifileucel (Amtagvi), a commercially manufactured autologous TIL product, marks a key milestone in integrating advanced therapy medicinal products (ATMPs) into routine care for solid tumours. Health Canada has since approved Lifileucel, with regulatory and funding decisions across the UK and Europe still pending. In this Perspective, we review the evidence base and outline key considerations for national adoption of TIL therapy. Despite promising results from clinical trials, TIL therapy requires complex coordination, including patient selection, tumour procurement, manufacturing logistics, lymphodepletion, and IL-2 administration; all contingent on specialised infrastructure and well-considered integrated care pathways. While commercial centralisation may ease logistical barriers, the high cost of TIL therapy necessitates careful health economic evaluation. A nationally coordinated effort is required to harmonise clinical prioritisation strategies, maintain oversight by multidisciplinary specialist tumour boards, and consider investment in future-proof decentralised manufacturing capacity. Collaborations and peer support such as through the Advanced Therapy Treatment Centre (ATTC) Network will facilitate phased, experience-led rollout with equity-focused service design.

## Background

After decades of research into harnessing tumour-infiltrating lymphocytes (TIL) as a therapeutic strategy, the United States (US) Food and Drug Administration (FDA) approved the first TIL therapy, Lifileucel (Amtagvi), in February 2024 for patients with advanced melanoma refractory to anti-PD-1 immunotherapy and, in the setting of BRAF V600 positivity, targeted therapy [1]. This was supported by data from the phase II C-144-01 study, which demonstrated an initial objective response rate (ORR) of 31.4% [2]. Further follow up at five years has confirmed durability of responses in 31.3% of treated patients with a median duration of response of 36.5 months [3], and several responses that deepened over time. In contrast, best estimates of durability of response with combination immunotherapy after previous anti-PD-1 exposure approximate 6-16.6 months [4], despite relative equivalence in the rate of initial responses [5–7]. Similar results have also been observed with academically manufactured TIL products, such as the Dutch and Danish product TM001 within the context of the first phase III randomised controlled trial [8]. These outcomes represented a breakthrough in a population with limited options, poor prognosis and therefore a high unmet need. Consequently, TIL therapy is currently under review by regulators and funding bodies outside of the US and Canada, including the United Kingdom (UK) Medicines and Healthcare products Regulatory Agency (MHRA) and the European Medicines Agency (EMA), both of which anticipate publishing decisions in 2026.

Should TIL therapy be approved for use in the UK and Europe, both patient and clinician uptake are expected to rise rapidly. Therefore, there is an urgent need for strategic service planning and guidance for integrating this novel therapy into standard clinical practice. This Perspective provides an overview of the evidence base for TIL therapy in melanoma and other solid tumours, evaluates its prospective role in treatment pathways, and outlines key barriers and opportunities for implementation alongside other Advanced Therapy Medicinal Products (ATMPs) both in the UK and broader European context.

## Evidence supporting TIL therapy

TIL therapy is a complex process, involving multiple steps: tumour harvest, TIL isolation and expansion, pre-infusion non-myeloablative lymphodepletion chemotherapy, and post-infusion high-dose interleukin-2 (HD-IL-2) to support in vivo survival and/or expansion of infused cells. Steven Rosenberg and colleagues at the US National Institutes of Health (NIH) were pioneers in the therapeutic use of TILs over four decades ago, and were the first to report on a large case series of 86 consecutive patients with advanced melanoma undergoing TIL therapy [9]. This study delivered two cycles of TILs and HD-IL-2, in combination with a single dose of pre-infusion cyclophosphamide lymphodepletion in 66% of treated patients [9]. Results demonstrated a slight, but non-significant benefit in the cyclophosphamide treated patients (ORR 35% compared to 31%) and improved outcomes for patients receiving TILs that were younger (i.e. spent less time in vitro), demonstrated shorter doubling times or exhibited high levels of lysis against autologous tumour targets [9].

Subsequent studies supported an association between improved responses and intrinsic TIL properties such as selection from younger cell cultures (*P* = 0.03), greater autologous tumour lysis (*P* = 0.02), higher numbers of TILs infused (*P* = 0.0003), increased proportions of CD8^+^ T cells within cultures (*P* = 0.001), shorter doubling times (*P* = 0.05), and cytokine secretion following autologous tumour stimulation (*P* = 0.04) [10, 11]. Early work identified GM-CSF as one such cytokine; however, this has since been superseded by more specific markers of tumour reactivity, including de novo production of INF-γ and TNF-α [12], and upregulation of the co-stimulatory receptor CD137, an anti-apoptotic activation marker [13]. These findings encouraged selection of minimally cultured, younger TILs with characteristics of tumour reactivity and high avidity for melanoma antigens [14, 15], although the requirement for testing of tumour reactivity was later eliminated from manufacturing to streamline the process, improve rates of successful TIL generation and reduce batch-to-batch variability [11, 16, 17]. Besides intrinsic TIL properties, the site of tumour harvest has been variably associated with differences in response and success of TIL generation. Early studies reported higher yields and response rates from subcutaneous compared to nodal sites (*P* = 0.006) [9, 10, 18]. However, subsequent analyses have not consistently confirmed these associations, showing no significant differences in response [19, 20]. Findings for TIL generation also remain mixed with the phase III M14TIL trial suggesting improved manufacturing success from nodal tissue (*P* = 0.037) [21], whereas other studies have demonstrated no difference in mean fold expansion by harvest site [22].

Pre-infusion lymphodepletion enhances TIL efficacy by depleting regulatory T cells, altering homeostatic cytokines, activating antigen presenting cells, and causing stimulation of lymphocytes [23]. Initially undertaken with cyclophosphamide monotherapy, the addition of fludarabine by Dudley et al. demonstrated further improvements in objective response from a median of 27.5% to a median of 45% (response defined as a ≥ 25% tumour reduction with no new lesions) [14]. Toxicities were predominantly haematologic, consisting of profound but transient lymphopaenia, neutropaenia and thrombocytopaenia, with 80% of patients (12/15) requiring transfusional support. Attempts to increase the depth of pre-infusion lymphodepletion included the addition of total body irradiation [24, 25]. Although responses increased, this was attenuated by increased toxicity from lymphodepletion and the need for stem cell rescue introduced additional complexity to an already demanding regimen [23, 24, 26]. Currently, the standard regimen for pre-infusion lymphodepletion remains the combination of cyclophosphamide and fludarabine.

IL-2 is a critical adjunct to TIL therapy, improving rates of response and in vivo T cell persistence [14, 27]. However, its administration is associated with significant toxicity, particularly at high doses, prompting efforts to explore de-escalation strategies such as decrescendo dosing and subcutaneous delivery [28–30]. Although cumulative IL-2 exposure does not consistently correlate with outcomes [10], high dose IL-2 (600,000-720,000 IU/kg) appears more effective than lower doses in some studies [27, 28], and at least one dose is now considered necessary to optimise TIL persistence. Despite these efforts, intravenous delivery remains standard, with regimens such as Lifileucel recommending six doses at 8-12 hourly intervals [31].

TIL-associated toxicity is generally acute and linked to lymphodepletion and HD-IL-2 [32, 33]. The rate of grade 5 toxicity is low, although a proportion of patients require intensive supportive care [34]. Unlike immune checkpoint inhibitors, autoimmunity is not a feature of TIL therapy, although these patients have frequently been excluded from trial participation with select reports of uveitis, skin depigmentation and audiovestibular dysfunction considered autoimmune in aetiology [23, 35]. Even so, the C-144-01 study allowed entry of patients with previous immune-related adverse events (irAEs), with no recurrence of irAEs observed following TIL administration [31].

**Fig. 1:**
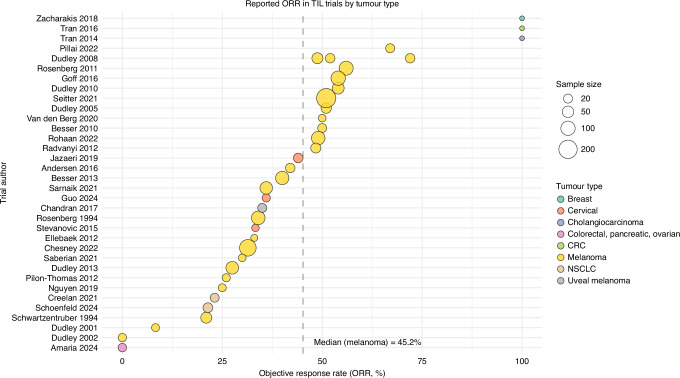
Bubble plot of objective response rates for TIL monotherapy, by tumour type and size of included participants. Trials are listed by first author and year (y axis) and objective response rate (ORR, %) (x axis). Bubble size is based on trial sample size, with tumour type by colour; CRC colorectal cancer, NSCLC non-small cell lung cancer.

Most available data derive from studies in melanoma, primarily cutaneous melanoma, with only a small number of mucosal melanoma cases included in the phase II C-144-01 trial, and a single phase II study of 20 patients receiving TILs for uveal melanoma [36–39]. Evidence has been summarised in a number of high-quality meta-analyses [34, 40]. TILs have also been investigated in other solid tumours including cervical, colorectal, cholangiocarcinoma, non-small cell lung, and breast cancers, though with variable efficacy and generally smaller sample sizes. Thirty-five TIL monotherapy studies conducted between 1994 and 2025 recruited 1463 patients, with a median of 23 patients per trial. The median objective response was 40% (range 0-72%) (Fig. 1). A further seven recent studies have combined TILs with other agents, including anti-PD-1 agents (nivolumab or pembrolizumab), oncolytic viruses, dendritic cells, BRAF/MEK inhibitors, and interferon-alpha for refractory melanoma (Table 1). Several of these combinations have a strong biological rationale, mediating TIL expansion, activation or infiltration (as in the case of dendritic cell vaccination and BRAF inhibitors), or preventing immunosuppressive interactions between tumour and TILs via the PD-(L)1 axis [41]. Combination therapy with anti-PD-1 agents represents the most advanced area of investigation, although data remain limited by small sample sizes and heterogeneity in treatment lines [42–44]. In a cohort treated with pembrolizumab and Lifileucel in the first-line setting, the ORR was 63.6% (14/22; 5 complete and 9 partial responses) [45], supporting further evaluation of this combination in a phase III trial. The phase III study, TILVANCE-301 [NCT05727904], will randomise patients with previously untreated advanced melanoma to receive Lifileucel in combination with pembrolizumab versus Lifileucel alone. Notably, patients assigned to the monotherapy arm will be permitted to cross over to Lifileucel following progression [46].

**Table 1:** Objective response rates and pertinent trial data for TIL combinations.

Author	Year	NCT	Tumour	Combination	Phase	Line	*N*	ORR (%)
Creelan	2021	NCT03215810	Non-small cell lung cancer	Nivolumab	I	2+	13	23.1
Huang	2022	NCT04443296	Cervical	Weekly cisplatin + radiotherapy	I	1	12	75
König	2024	NCT04165967	Cutaneous melanoma	Nivolumab	I	2+	9	22
Kverneland	2021	NCT03296137	Agnostic	Ipilimumab + nivolumab	I/II	2+	25	20
L’Orphelin	2024	2015-005066-31 (EudraCT)	Cutaneous melanoma	Nivolumab	I/II	1	4	75
Monberg	2025	NCT04217473	Mucosal melanoma	TILT-123	I	2+	17	11.7
Nielsen	2023	NCT03725605	Soft tissue sarcoma	LTX-315	I	2+	4	0
O’Malley	2021	NCT03645928	Cervical	Pembrolizumab	II	1	10	50
O’Malley	2021	NCT03645928	Head and neck squamous cell carcinoma	Pembrolizumab	II	1+	14	42.9
O’Malley	2021	NCT03645928	Melanoma	Pembrolizumab	II	1+	8	87.5
Saberian	2021	NCT00338377	Cutaneous melanoma	Dendritic cell vaccination (pulsed with MART-1)	II	2+	8	50
Sarnaik	2015	NCT01659151	Cutaneous melanoma	Vemurafenib	II	1+	12	42
Thomas	2024	NCT03645928	Cutaneous melanoma	Pembrolizumab	II	1	22	63.6
Verdegaal	2020	NCT03638375	Cutaneous melanoma	IFN-alpha	I/II	2+	34	29
Wang	2020	-	Osteosarcoma	Nivolumab	-	3+	30	33.3
Zackarakis	2018	NCT01174121	Breast	Pembrolizumab	II	2+	1	100

The approval of Lifileucel by the FDA was based on the C-144-01 phase II trial [NCT02360579] of 153 patients, expanding on an earlier 66-patient cohort [2, 31]. Enrolled patients were heavily pre-treated with a median of three prior lines of therapy, over 80% of whom had previously received dual checkpoint inhibition [2, 31]. Despite poor prognostic factors such as heavy disease burden and an elevated LDH, the ORR was 31.4% (48/153; 8 complete responses (5.2%)), reinforcing data from earlier studies in patients with bulky disease and brain metastases [2, 25]. At 5 year follow up, the median duration of response was 36.5 months, with 31.3% of responders completing 5 year follow up with ongoing disease response [3].

The only phase III study [M14TIL/NCT02278887] of TIL therapy assessed an academic TIL product (TM001) from the Netherlands Cancer Institute and Herlev Hospital (National Centre for Cancer Immune Therapy, Denmark; CCIT-DK) with 1:1 randomisation against ipilimumab, delivered at a dose of 3 mg/kg every 3 weeks for a maximum of 4 doses [8]. Recruitment spanned nearly eight years from September 2014, and the choice of control arm reflects the contemporary standard of care at the time the protocol was finalised. Most of the 168 participating patients had received prior anti-PD-1 in either the adjuvant (24%) or first-line metastatic setting (62%). While inclusion of patients treated in the first-line metastatic setting was allowed, ultimately only 11% of patients had received no prior systemic therapy and only 14% had not received prior anti-PD-1. Clear benefit was demonstrated for the TIL group, with an ORR 49% (41/84; 17 complete responses (20.2%)) compared to 21% with ipilimumab and a 44% reduction in the risk of progression or death (HR 0.56, 95% CI 0.39–0.79). Notably, lower rates of serious AEs were observed in the TIL group (15% vs 27%), together with a higher overall health-related quality of life. Consequently, this trial reinforced the role for TIL in the post-anti-PD-1 metastatic setting where options are limited and responses to other therapies remain modest. Crucially, it also supported eventual access to TIL for Dutch and Danish patients with anti-PD-1 refractory disease through basic insurance coverage [47].

In guidelines released by the European Society of Medical Oncology (ESMO), aligned with those of the National Institute for Health and Care Excellence (NICE), first-line immunotherapy options for advanced melanoma include anti-PD-1 agents pembrolizumab or nivolumab, either as monotherapy or in combination with ipilumumab or relatlimab [48, 49]. Targeted therapy with encorafenib/binimetinib or dabrafenib/trametinib is also available for the ~50% of patients with a BRAF V600 mutation. While sequencing between these agents is flexible, nuances exist depending on duration of prior treatment, adjuvant exposure, and tolerability. The advent of combination immunotherapies such as ipilimumab + nivolumab and nivolumab + relatlimab has transformed the treatment landscape, and there is now no universally accepted second-line immunotherapy option for patients progressing after first-line therapy. For most, participation in a clinical trial or retreatment in selected cases represents the primary therapeutic pathway.

The MHRA application for Lifileucel, mirroring that of the FDA, applies specifically to previously treated, unresectable or advanced melanoma, thereby positioning TIL therapy after at least one line of anti-PD-1 treatment. Some data suggest higher response rates to TILs in anti-PD-1-naïve patients (ORR 56% vs 24% [50]). In real-world reports of Lifileucel, this benefit appears to extend to later-line settings, with an ORR of 60.9% among patients who had received two or fewer prior therapies compared with 33.3% in those with three or more [51]. Conversely, subsequent checkpoint blockade following TIL therapy has shown limited activity, with an ORR of only 7.2% (7/97, 2 CR, 5 PR) [52]). Further evidence is needed to clarify the efficacy of checkpoint inhibition after TIL therapy.

Treatment options remain limited across Europe and the UK for patients with advanced melanoma who progress following adjuvant anti-PD-1 therapy. Regulatory constraints and longer approval timelines under the EMA and MHRA compared with the FDA have also contributed to delays in access to novel therapies [53]. Many of these agents, including T-cell engagers and other immunotherapies, require frequent hospital visits and complex dose-escalation schedules. In this context, TIL therapy represents an appealing option, particularly given its single-infusion administration. Moreover, it carries a lower risk of irAE recrudescence. Although non-myeloablative lymphodepletion and HD-IL-2 are associated with distinct toxicities, these are generally acute, predictable, and transient, with performance status typically recovering to baseline within weeks of treatment. Notably, the C-144-01 trial reported no recurrence of prior irAEs in patients previously treated with checkpoint inhibitors [31], even with trial eligibility requiring patients to taper steroids to physiological levels to minimise lymphotoxicity on infused TILs [54]. Collectively, these factors position TIL as a potentially safer option for patients with previous severe irAEs, especially when re-treatment with checkpoint inhibitors may be contraindicated.

Patient advocacy groups are also aligned with benefits attendant with access to TILs, with UK-based patient advocacy group *Melanoma Focus* expressing support for its approval and access [55], and patient and public involvement central to the Horizon Europe funded PragmaTIL project [56].

## Regulatory landscape

The FDA approved Lifileucel (LN-145) in February 2024 for patients with advanced melanoma refractory to anti-PD-1 immunotherapy [1]. Health Canada followed in August 2025 with a conditional approval issued under a Notice of Compliance with conditions, requiring submission of further confirmatory trial data [57]. A marketing authorisation for the same indication has been submitted to the MHRA and a technical appraisal to inform NHS funding decisions by the National Institute of Health and Care Excellence (NICE) (ID3863) is similarly pending [58]. In Europe, a marketing authorisation application for Lifileucel was withdrawn from the EMA in July 2025 [59], effectively halting the near-term prospect of commercial access to TIL therapy and leaving patients reliant on clinical trial or academic manufacturing pathways. This poses significant challenges, particularly given the absence of a defined EMA pathway for approval of academic rather than commercial ATMPs. In contrast, post-Brexit changes to MHRA processes have prioritised greater speed and flexibility, including the introduction of the International Recognition Procedure (IRP) [60], which leverages assessments from trusted regulators such as the FDA to enable potential 60-day approvals. The Innovative Licensing and Access Pathway (ILAP) offers a complementary mechanism, fostering collaboration between developers, regulators, and the NHS to accelerate patient access to novel therapies and bridge the academic-commercial gap for cellular therapies [61].

Similar to the recent roll out of chimeric antigen receptor T-cell (CAR-T) therapies for patients with haematological malignancies in the UK, it is anticipated that dedicated NHS TIL therapy hubs will be established if a favourable NICE appraisal decision can be achieved [62]. Indeed, a national tender process for service provision was re-activated in September 2025. A comparable, experience-led implementation will likely be required across Europe, where access to TIL therapy currently remains largely confined to academic trial centres with established TIL infrastructure. Outside of the Netherlands and Denmark, where participation in the M14TIL trial has facilitated in early access programmes and limited clinical use under hospital exemptions authorised by national competent authorities, routine access across Europe remains highly restricted [47].

## Cost-effectiveness of TIL therapy

The economic viability of TIL therapy must be considered in light of its substantial production and delivery costs. However, these upfront expenses may be offset when compared to the cumulative cost of checkpoint inhibitors, which are often administered for two years or more and incur additional costs related to the management treatment-related toxicities. Comparatively, TIL therapy is delivered as a one-time treatment, potentially reducing long-term expenditure.

Currently, immunotherapy in the ‘palliative’ setting accounts for 68% of total drug acquisition costs for melanoma treatment in England [63], highlighting the significant financial burden of prolonged metastatic care. These costs vary based on follow up intensity, inpatient care requirements, treatment duration, and the proportion of patients receiving immunotherapy [64, 65]. For patients who achieve durable long-term survival, monthly per-patient costs were found to be lower in a pooled cost-of-illness analysis undertaken across the UK, Italy and France [66]. This suggests that the high up-front cost of TIL may be justified if it leads to durable responses and eliminated the need for ongoing systemic immunotherapy. Table 2 outlines the NHS-indicative pricing for treatments available in the advanced melanoma setting beyond first line, as listed in the British National Formulary. It should be noted that most of these treatments are accessed via commercially confidential discounts [63].

**Table 2:** NHS indicative pricing and estimated cumulative costs for approved agents in the metastatic setting.

Agent	NHS indicative price	Estimated course
Dabrafenib/trametinib	**₤1400**/28 × 75 mg **₤1120**/7 x 2 mg	₤131,040/year
Encorafenib/binimetinib	**₤1400**/42 x 75 mg **₤2240**/28 x 45 mg	₤131,040/year
Ipilimumab	**₤15,000**/200 mg	₤60,000 (4 cycles)
Ipilimumab/nivolumab	**₤3750**/50 mg **₤2633**/240 mg	₤25,532 (4 cycles)
Nivolumab	**₤5266**/480 mg	₤126,384 (24 cycles)
Nivolumab/relatlimab	**₤6,134.75**/240 mg/80 mg	₤127,234 (24 cycles)
Pembrolizumab	**₤10,520**/400 mg	₤182,346.66 (2 years)

To further explore the economic viability of TIL therapy, Ten Ham et al. conducted a cost-effectiveness analysis of the Danish-NKI TIL product compared with ipilimumab [67]. Using lifetime undiscounted costs alongside estimates of life years and quality-adjusted life years (QALYs), they estimated a cost-saving of €86,466 ( ~ ₤72,982) per patient in the Netherlands and €98,826 ( ~ ₤83,414) in Denmark, concluding that TIL was cost-effective compared to ipilimumab [67]. This analysis assumed a total upfront expenditure of €117,940 ( ~ ₤103,085) per patient for TIL, inclusive of assessments and supportive care [67]. However, the study was limited by the duration of follow-up, and approximately 20% of patients received subsequent ipilimumab upon progression after TIL [8]. Moreover, these estimates reflect a non-commercial product. The commercial alternative, Lifileucel, carries a significantly higher cost, with a list price of $515,000 ( ~ ₤381,640) in the United States for the product alone [68]. Given the variation in melanoma costs across Europe [69], formal health economic analyses are needed in other European contexts to assess the full spectrum of associated costs, including infrastructure, workforce, logistics and long-term patient management.

Beyond direct treatment costs, productivity loss from advanced melanoma is estimated at €217.1 million (₤234.58 million) annually across Europe and the UK [69]. With melanoma incidence expected to rise, the financial burden will likely increase, reinforcing the need for investment in effective therapies that may reduce long-term costs [64]. While centralised processing from a commercial supplier offers convenience and helps overcome access barriers, the long-term affordability of such products remain questionable. As demand grows, driven by clinician familiarity and expanded site-based delivery following MHRA and EMA approvals, there is a compelling case for investing in onshore TIL manufacturing as a means to mitigate costs and improve access to care.

## Implementation readiness

While TIL therapy is now clinically validated, scaling delivery nationwide requires dedicated infrastructure and experience. A small number of UK and European sites have already participated in industry-sponsored TIL trials such as C-144-01, TILVANCE-301 [NCT05727904 [46]] and IOV-LUN-202 [NCT04614103 [70]], alongside academic trials held at centres such as The Christie in Manchester [71], University Hospital Basel [42], the Netherlands Cancer Institute [8, 72] and the National Centre for Cancer Immune Therapy in Denmark [8]. These institutions followed protocols broadly aligned with those of the NIH and Lifileucel, collectively forming a foundation of institutional knowledge that can be leveraged in future rollout.

Wider adoption of TIL therapy can benefit from existing infrastructure for advanced therapies, notably CAR-T. In England, twenty adult and three paediatric centres are currently authorised for CAR-T delivery with implementation following a phased, experience-led model [73]. These centres are integrated through the Advanced Therapies Treatment Centres (ATTC) Network which, in concert with the Cell and Gene Therapy Catapult, aims to enhance access, streamline delivery, and foster UK readiness for innovation [74, 75]. This ‘hub and spoke’ approach, built on the previously established process for haematopoietic stem cell transplantation and has been mirrored in CAR-T rollout in other European centres [76].

Successful CAR-T implementation relied on support from the European Society for Blood and Marrow Transplantation (EBMT), which provides post-marketing safety monitoring and coordinates Joint Accreditation Committee of the International Society for Cell & Gene Therapy and EBMT (JACIE) accreditation of delivering sites [77]. JACIE accreditation standards encompass delivery of immune effector cells with a broad designation that includes diverse manufacturing methods, constructs, safety profiles and indications. Tumour-infiltrating lymphocytes fall within this broad classification, and the existing framework could readily be applied or adapted to provide accreditation and oversight for TIL therapy. Similar accreditation and oversight mechanisms could be adapted for TIL therapy. Additionally, national tumour boards, already used for other purposes such as genomic reviews and trial matching [78], could be repurposed to assist with patient selection, multidisciplinary communication, and streamlining of complex processes.

A key challenge in TIL therapy provision is streamlining its multi-step process [79]. Bottlenecks include timely tumour procurement, scheduling of lymphodepletion and IL-2 administration, and coordination of manufacturing within a tight 22-day production window for Lifileucel. Without appropriate patient selection, disease progression may lead to attrition and increased surgical risk, despite generally low morbidity [21, 22, 31, 80]. Emerging real-world data on Lifileucel use indicate an attrition rate of approximately 10% due to disease progression, a figure that may be reduced through workflow optimisation [81] and central coordination by a dedicated cellular therapy nurse [82]. These logistical challenges are compounded by national disparities in digital systems, scheduling capacity and governance processes including variation in interpretation and implementation of tissue legislation such as the Human Tissue Act 2004 and its European counterpart Directive 2004/23/EC.

To support broader adoption, expansion of capabilities across both central and regional delivery sites is also essential. Currently, expertise in administering ATMPs such as CAR-T is predominantly concentrated among haematologists. As tissue-engineered therapies increasingly enter solid tumour treatment pathways, cross-specialty training, particularly for medical oncologists, is critical. Establishing structured, shared learning opportunities, for example through dedicated preceptorships and symposia, will also be key to managing complex patient cases and fostering multidisciplinary collaboration.

## Future opportunities for TIL

While Lifileucel marks a major milestone in cellular therapy for solid tumours, it should be viewed as an initial step forward rather than a destination. Substantial risk is involved in building an entire national service around a single commercial product, particularly one currently located at an off-shore, US-based site. Furthermore, innovation in this space is accelerating, with research efforts exploring ways to improve upon polyclonal TILs, including reintroduction of selection for tumour-reactivity, as well as neoantigen specificity, phenotypic profiling, and ex vivo enhancement through engineering [83]. Ongoing trials are also evaluating combination regimens [84] and use in different treatment settings, including the first line [46]. Emerging data suggest that earlier administration of TIL therapy may yield more durable responses while reducing the risk of overtreatment and chronic toxicities associated with prolonged checkpoint inhibition. The ‘one-and-done’ approach in the first-line setting offers the additional advantages of extending treatment-free intervals and reducing attrition from disease progression in later lines. However, the design of TILVANCE-301 will not definitively address this question and limits the interpretation and generalisability of its findings for clinical practice. The use of pembrolizumab as the control arm would not be considered standard of care for the poorer-prognosis population in whom TIL therapy is typically indicated [85, 86], while conversely, the toxicity of TIL therapy raises concern when applied to patients for whom single-agent PD-1 therapy remains appropriate. Moreover, the combination of pembrolizumab with TIL therapy represents an escalation in treatment intensity rather than testing the ‘one-and-done’ concept central to TIL therapy, while also obscuring attribution of efficacy to the TIL product itself.

Head-to-head comparisons with contemporary combination immunotherapies remain essential to define the optimal sequencing and positioning of TIL therapy within evolving treatment paradigms. In parallel, efforts to mitigate treatment-related toxicities include the evaluation of modified lymphodepletion regimens [87] or substituting high-dose IL-2 with agents such as IL-2 fusion proteins [88], pegylated IL-2 [89], IL-2Rβγ agonists [90], or other cytokines including IL-7 and IL-15 [91]. Considering the frequent divergence between patient priorities and conventional trial endpoints, strong academic leadership will be essential to ensure that study design and implementation remain driven by clinical relevance and patient benefit rather than regulatory or commercial imperatives [92, 93].

To support these developments and reduce long-term costs, investment in onshore GMP manufacturing will be crucial. This is especially important given the high costs associated with commercial products like Lifileucel and the need to build an adaptable, resilient, and scalable system that can respond to new indications and technologies. Domestic production capability would not only mitigate reliance on overseas suppliers but also enable countries to participate in and lead next-generation TIL research and innovate independently of commercial constraints. This would include meaningful contribution to pan-European initiatives such as the PragmaTIL project [56], which aims to optimise TIL therapy delivery across tumour types through harmonised production, trial frameworks and access strategies. This project is currently active across academic hospitals in Spain, Denmark, the Netherlands, France, Israel and Sweden. Engagement in such consortia positions national systems at the forefront of global oncology innovation, enabling them to shape emerging standards in cellular therapy, accelerate translational development and ensure that patients benefit from the next generation of engineered and personalised TIL products.

## Summary

TIL therapy represents a promising emerging option in second-line treatment of advanced melanoma, after failure of checkpoint inhibitor therapy. Despite the complexity of its delivery, the potential for durable remission positions it as a compelling alternative to immune checkpoint inhibitors, especially in a landscape lacking clear consensus on second-line treatment. Although formal cost-effectiveness analyses are still needed, the single administration ‘one-and-done’ approach may ultimately offer economic and practical advantages.

The outlook for TIL therapy is likely to improve further with strategic investment in decentralised GMP manufacturing facilities, which would serve to future-proof TIL access and enhance scalability, particularly in the event of proven success across other tumour types.

To ensure safe and effective implementation of Lifileucel, a phased, experience-led approach to roll-out is essential. This should be supported by specialist clinical networks, and coordinated centrally through multidisciplinary TIL tumour boards, which can provide insight and facilitate best practices.

## Data Availability

All data analysed are publicly available, as referenced respectively. Further information is available from the corresponding author upon reasonable request.
